# Feature Selection Has a Large Impact on One-Class Classification Accuracy for MicroRNAs in Plants

**DOI:** 10.1155/2016/5670851

**Published:** 2016-04-12

**Authors:** Malik Yousef, Müşerref Duygu Saçar Demirci, Waleed Khalifa, Jens Allmer

**Affiliations:** ^1^Computer Science, The College of Sakhnin, 30810 Sakhnin, Israel; ^2^The Institute of Applied Research, The Galilee Society, P.O. Box 437, 20200 Shefa Amr, Israel; ^3^Molecular Biology and Genetics, Izmir Institute of Technology, Urla, 35430 Izmir, Turkey; ^4^Bionia Incorporated, IZTEKGEB A8, Urla, 35430 Izmir, Turkey

## Abstract

MicroRNAs (miRNAs) are short RNA sequences involved in posttranscriptional gene regulation. Their experimental analysis is complicated and, therefore, needs to be supplemented with computational miRNA detection. Currently computational miRNA detection is mainly performed using machine learning and in particular two-class classification. For machine learning, the miRNAs need to be parametrized and more than 700 features have been described. Positive training examples for machine learning are readily available, but negative data is hard to come by. Therefore, it seems prerogative to use one-class classification instead of two-class classification. Previously, we were able to almost reach two-class classification accuracy using one-class classifiers. In this work, we employ feature selection procedures in conjunction with one-class classification and show that there is up to 36% difference in accuracy among these feature selection methods. The best feature set allowed the training of a one-class classifier which achieved an average accuracy of ~95.6% thereby outperforming previous two-class-based plant miRNA detection approaches by about 0.5%. We believe that this can be improved upon in the future by rigorous filtering of the positive training examples and by improving current feature clustering algorithms to better target pre-miRNA feature selection.

## 1. Introduction

MicroRNAs (miRNAs) are short regulatory nucleotide sequences which were discovered about 2 decades ago [1]. Since then, they were shown to exist in organisms ranging from sponges [2] to human [3] and also in plants where they may have evolved independently [4]. Due to their involvement in posttranscriptional regulation, miRNAs have been implicated in human diseases [5, 6] and, for example, in plant stress response [7]. Regulation of gene expression is of great interest and, therefore, miRNAs have received increasing interest.

Some miRNAs have been experimentally detected and they are stored in databases such as miRBase [8] and miRTarBase [9]. Unfortunately, experimental detection of miRNAs is quite involved and further suffers from the fact that some miRNA-target combinations may be expressed only in response to specific external or internal stresses so that it seems impossible to experimentally detect all miRNAs of any higher eukaryotic organism [10–13]. Additionally, current approaches may not use the full potential of available experimental techniques [14]. Even among the experimentally validated miRNAs in miRBase, there may be entries which are dubious and we have shown that some may not represent true miRNAs [15]. Thus, computational methods for miRNA detection are required to complement experimental approaches.

Computational approaches for miRNA detection and miRNA's target detection [16] are generally based on machine learning [17]. In order to use miRNAs in machine learning, they must be parameterized and many different features have been described [18]. For successful machine learning, known examples need to be provided to the algorithms and these are generally collected from miRBase. Except for a few examples [10], two-class classification is used for machine learning. In this case in addition to the positive learning examples, negative ones have to be provided.

Unfortunately, the negative class cannot be defined for any larger eukaryotic organism since any part of a genome must be coexpressed with any other part of the genome to prove that they cannot interact as miRNAs, a futile endeavor. Therefore, examples for the negative class have been arbitrarily created in studies performing machine learning for miRNA detection, but the most abundantly used negative dataset is from [19] although new datasets are being proposed [20]. While in our experience the dataset is a significant improvement over using randomly selected or generated examples, it is likely to also contain positive examples.

To overcome the problem of needing to define artificial negative examples, one-class classification can be used instead [21, 22]. One-class classification (OCC) only needs the target class for learning and then classifies unknown examples into target or unknown (outlier) class. We have recently analyzed the use of one-class classification for miRNA detection in plants and found that it is competitive in comparison to two-class classification [23].

We also found that some features among the hundreds of features described for miRNA parameterization [24] are more discriminative than others. Unfortunately, feature selection is NP-hard [25] and selecting the best subset from more than 700 features on a per-dataset basis is not possible in an acceptable amount of time. Apart from the computational complexity of feature selection, methods have only been well established for two-class classification [26–28], while only few approaches deal with one-class classification [29–31].

In this study, we used different feature selection approaches and evaluated the effectiveness of each one against the others for the classification of miRNAs from different plant species using one-class classification. We found that clustering of features is important for classification performance and helps to improve classification accuracy by up to 36% (*Oryza sativa*) and on average by about 30% compared to selecting features by the lowest information gain. Clustering in its present form, however, is ill suited for selection from pre-miRNA features, since logically correlated features may be uncorrelated when examining the feature vectors in isolation and vice versa. Therefore, the clustering methods performed best, but not the expected clustering method which selected the feature with the highest information gain from each cluster. With a peak performance of 98.80% accuracy for* Zea mays*, feature selection using clustering methods was able to increase the accuracy achieved in our previous studies by 1–4% and the two-class classification benchmark PlantMiRNAPred by ~0.5%. This study showed that feature selection is effective despite varying data quality. Concluding, it seems important to devise effective data selection methodologies to ensure that only true miRNAs are used as positive examples. Novel feature clustering approaches taking into account logical feature correlation need to be coupling this data filtering strategy. When both are achieved, one-class classification may significantly outperform two-class classification for pre-miRNA detection in all cases (for plant) since the gap is already closed with this study.

## 2. Materials and Methods

### 2.1. Data

We downloaded all available microRNAs from selected plant species from miRBase [8] (Releases 20 and 21). The selected species were* Glycine max* (gma),* Zea mays* (zma),* Sorghum bicolor* (sbi),* Physcomitrella patens* (ppt),* Arabidopsis thaliana* (ath),* Populus trichocarpa* (ptc), and* Oryza sativa* (osa). Our negative data pool consisted of the 980 pseudo pre-miRNAs constituting the PlantMiRNAPred dataset [32]. For these data, all pre-miRNA features were calculated as described previously [24, 33, 34].

### 2.2. One-Class Classification

One-class classification was performed using randomly sampled 90% of the positive data for training and 10% for testing (Supplementary File 1 in Supplementary Material available online at http://dx.doi.org/10.1155/2016/5670851, Figure 2). In addition to the 10% positive data during testing, the pseudo negative sequences were used as unknown class. To determine the stability of the feature set, the process was repeated 100 times. We used the DDtools [35] implementation of a OCC.

### 2.3. Feature Selection Strategies

Feature selection is not a straightforward process since two features which individually may not be very discriminative may have high selective power in combination. Additionally, we have more than 700 features so trying all combination of features from 1 to 700 for all datasets considered in this study is not possible. In order to test the impact of feature selection on the classification accuracy, four negative and four positive feature selection methods were designed. According to our previous research [24], 50 features seem sufficient for miRNA detection [24] and this limit was used in this study. Among the feature selection methods used in this study, only PCF is using only the positive class whereas all others are using both classes.

#### 2.3.1. Selecting Features with Low Information Gain (LIG)

Information gain was calculated on a per-dataset level using KNIME [36] and for each dataset the process had to be iteratively repeated until the 50 features with lowest information gain. First information gain was calculated and since most features received a score of zero they could not be differentiated. After removing all features with information gain above zero and recalculating information gain for the remaining features, the pool of features that receive zero became successively smaller until only 50 features remained. These remaining features were selected and used for classification (Supplementary File 1, LIG).

#### 2.3.2. Random Feature Selection (RFS)

50 features, from the overall list of features, were randomly selected for each dataset (Supplementary File 1, RFS).

#### 2.3.3. Selecting Random Feature from Feature Clusters (RFC)

Since correlated features may not much contribute to the discriminative power of a feature set, features were clustered into 100 clusters and from each cluster a random feature was selected from which 50 random features were selected for classification (Supplementary File 1, RFC).

#### 2.3.4. Selecting Features from Clusters (SFC)

Instead of selecting a representative feature per cluster (as in RFC and HIC), this approach selects up to three clusters and uses all features therein to see the impact of correlation among features on the classification (Supplementary File 1, SFC).

#### 2.3.5. Selecting Features with High Information Gain (HIG)

Contrary to the selection of features with low information gain, we selected the features with the highest information gain on a per-dataset basis (Supplementary File 1, HIG).

#### 2.3.6. Selecting Feature with the Highest Information Gain from Feature Clusters (HIC)

Among the 700 features describing miRNAs are likely some which describe very similar information and, to avoid overrepresentation of such information, it may be beneficial to cluster such features and use single features to represent each cluster. 100 clusters were produced per dataset and from each cluster the feature with the highest information gain was selected. The selected features were again ranked via information gain and the top 50 were selected (Supplementary File 1, HIC).

#### 2.3.7. Zero-Norm Feature Selection (ZNF)

For each feature vector, we define the zero-norm to be the nonzero values for all positive examples. First features whose vector consists of zero values are removed. Additionally, we defined #_a_(*v*) as the number of values with nonzero value, for example, #_a_(0.4,0, 0.6,0, 0,0.8,0, 1,1.4) = 5. Moreover, we define different thresholds for #_a_(*v*) to determine the relevance of a feature and remove the ones below a given threshold (Supplementary File 1, ZNF).

#### 2.3.8. Pearson Correlation-Based Feature Selection (PCF)

The Pearson correlation-based feature selection method was introduced by Lorena et al. [29]. The Pearson correlation measure allows detection of the linearity relation among features. The pairwise distances among features were calculated using Pearson correlation. Features with lower correlation were preferred during the feature selection process (Supplementary File 1, PCF).

## 3. Results and Discussion

### 3.1. Feature Selection

Feature selection is an important process in machine learning and not all of the more than 700 features which have been proposed to describe a pre-miRNA might be useful. We used eight feature selection methods and performed one-class classification using each of the selected feature sets. 50 features were used as a target and we did not try different sized feature sets. Additionally, for each feature selection method a combined feature set was created containing the most commonly selected features (denoted by suffix _comb). To judge feature selection success, one-class classification was performed using the selected features. The average accuracy of 100 times cross validation served as performance measure to evaluate the feature selection methods. The selected features by the different methodologies are presented in Supplementary Table 1.

**Figure 1:**
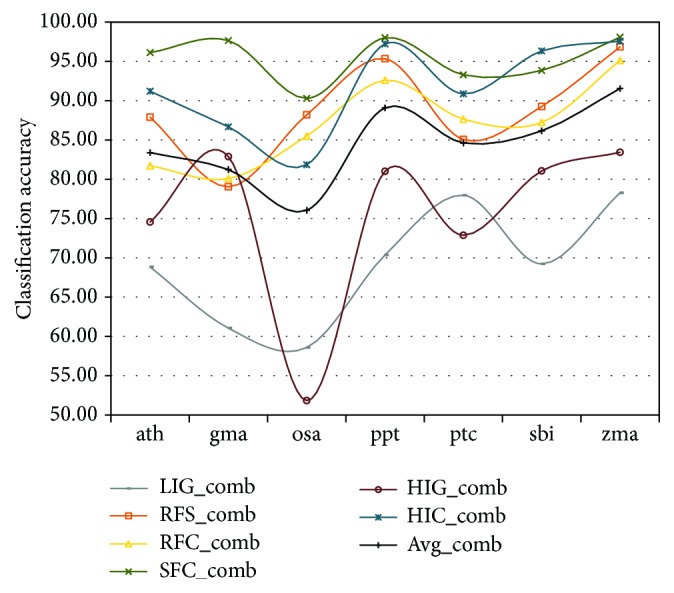
Classification accuracy of the combined feature sets on a per-organism basis. Note that there is no mathematical relationship that supports the connection among points (measurements) and that the lines were only added for visual guidance to enhance the synchronized variation on a per-organism basis. Supplementary Table 2 contains the underlying data (including sensitivity and specificity) and a plot for the individual feature selection on a per-species basis.

For the combined feature selection method, SFC and HIC outperform all other feature selection methods consistently for all datasets (Figure 1). However, the performance varies strongly with the organism and the variation is similar for all feature selection methods. This synchronized variation indicates that the available training data is either not sufficient or not accurate enough or both. This is in line with a previous study where we showed that not all data on miRBase may represent true miRNAs [15]. Nonetheless, since the feature selection methods SFC and HIC consistently achieved the highest accuracy compared to all other feature selection methodologies, the assessment of feature selection methods is not hampered by the shortcomings of the datasets. The average accuracy over all feature selection methods can further help define dataset quality. ppt has the best result (~88%) followed by zma (~87%), while gma (~80%) and osa (~78%) are tailing the list (Supplementary Table 2).

**Figure 2:**
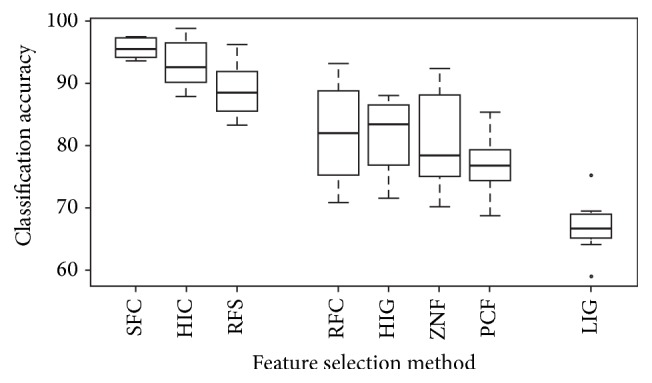
Accuracy distribution for the seven selected organisms assessed using one-class classification.

Data in Figure 1 is in respect to combined feature sets and it may be informative to investigate accuracy in respect to feature selection on a per-organism basis (Figure 2) since the average accuracy of average accuracies for all feature selected methods is slightly higher for individual feature selection (84.69%) as compared to combined feature selection (84.58); see Supplementary Table 2 for more information.

Figure 2 shows that the feature selection methods fall into three categories, improved accuracy (SFC, HIC, and RFS), no or little effect (RFC, HIG, ZNF, and PCF), and finally LIG, which was chosen as a negative control along with RFS and SFC. LIG which selects the features with the lowest information gain performs as expected and leads to the lowest effectiveness of the resulting classifiers. Interestingly, RFS and SFC which we expected to provide low performance lead to effective classifiers. Many feature clusters exist in the large feature space and RFS (random feature selection) likely picked from different clusters. This is supported by the observation that the spread of RFS is much larger than for SFC and HIC. SFC merely selects features from three clusters and was expected to have low performance. However, it turned out to be the best performing algorithm which we attribute to a critical problem in clustering for feature selection. We used *k*-means clustering on the feature vector containing calculations for positive examples. Unfortunately, features like % A and % G are strongly correlated although their vectors are anticorrelated. This is true for many of the features among the more than 700 described features and the opposite is also true where two feature vectors appear highly correlated although they describe logically uncorrelated information. For these reasons, HIC which selects the feature with the highest information gain from each cluster performed not as well as would be possible if correlation would be perfectly following logical constraints. RFC (random feature selection from clusters) performed better than HIG, ZNF, and PCF, which supports the notion of why HIC did not consistently show the best performance among all algorithms. Nonetheless, HIC showed the overall best performance for zma with 98.80% accuracy. Among the feature selection methods, there is a large performance gap (~38%). On a per-organism basis, the gap reaches ~36% (*O. sativa*) but on average it is close to 30% (Supplementary Table 2), which highlights the importance of proper feature selection.

### 3.2. Organism-Based Feature Selection

Feature selection as described above was performed on a per-organism basis and from the selected features the most common ones were used to define a combined feature selection (Supplementary Table 1). It was our belief that using feature selection on a per-organism basis would have an effect on the accuracy that can be achieved. On average this is true and a slight advantage of about 0.2% accuracy when comparing the averages of the averages of all feature selection methods can be observed (Supplementary Table 2). Unfortunately, it turns out that the number of positive results and their correctness are more important than the feature selection method and whether it is performed on a per-organism basis or not. For* A. thaliana*,* G. max*, and* O. sativa* the assumption holds but, for the other species used here, the results were not so clear. Perhaps the strategy of selecting the 50 most common features among the first 100 features into the combined feature set is not actually selecting a feature set that would describe the selected clade.

**Figure 3:**
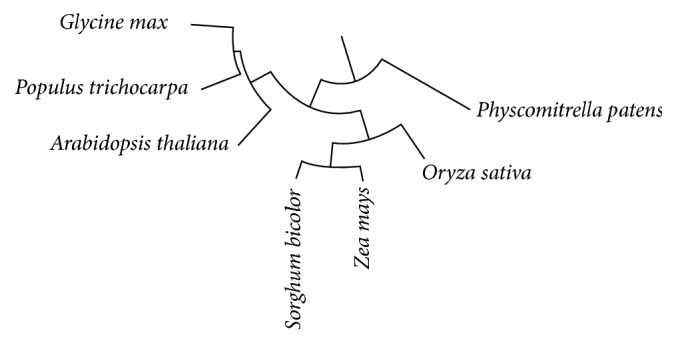
Phylogenetic relationship among the selected plant species.

In order to investigate whether feature selection has to be made on a per-species basis, we used the interactive tree of life [37] to establish the phylogenetic relationship among the selected plant species (Figure 3). Three larger groups are visible one of which consists of a single species (ppt) while the other two contain three species each.

We checked the deviation of the accuracy achieved using feature selection on a per-organism basis to the average for the organisms and found that for SFC and HIC the distance of* P. patens* to the average was different from the distances of the other organisms which were alike in their groups (Supplementary Table 2) as expected from their placement in the phylogenetic tree (Figure 3).

### 3.3. Performance Comparison

In a previous study we introduced sequence motifs as features for pre-miRNA detection and compared one-class classification to two-class classification [23]. Compared to the results we achieved with MotifmiRNAPred, this study shows that feature selection is essential for classification performance (Table 1).

For some of the organisms (gma, zma, and sbi), PlantMiRNAPred, which employs two-class classification, performs better than the one-class classification performed in this study. On the other hand, on average one-class classification is slightly better and performs better than PlantMiRNAPred on some organisms (ath, ppt, ptc, and osa). The improvement to MotifmiRNAPred which also uses one-class classification is about 3.5% on average and this again highlights the importance of feature selection. The improvement of classification accuracy in this study (using one-class classification) over PlantMiRNAPred (using two-class classification) is about 0.5% accuracy, but it is an important step since for the first time OCC based pre-miRNA detection outperforms two-class classification.

## 4. Conclusions

One-class classifiers were trained using different feature sets selected by various feature selection methods (see Supplementary Table 1 for selected features). Feature clustering seems essential since the best performing methods use clustering (Figure 2). On average 30% accuracy can be gained using feature selection (Supplementary Table 2). Compared to previously published methods, the OCC using SFC filtered features performs better (~3.5%) than our previously established OCC and slightly better (~0.5%, ~96%) than the best two-class classifier for plant pre-miRNA detection (Table 1).

A peak accuracy of close to 99% may seem sufficient for miRNA detection, but this figure needs to be put into context with millions of hairpins in a genome which could constitute a pre-miRNA. Thus accuracies above 99.99% (about 1000 false positives in a genome of 3Gb) are needed if all prediction ought to be tested experimentally.

To achieve such high accuracies, the following points need to be tackled:

1. In the future, it will be important to devise a method to filter the positive training data since not all datasets seem to represent a pure class and this strongly influences classification accuracy (Figure 1).
2. Clustering is essential for feature selection, but current clustering methods do not take into account logical correlation (Figure 2).
3. A switch to OCC needs to be performed since negative data for pre-miRNAs does not exist [10, 40].
4. Feature selection should be performed on a per-organism basis and how classifiers perform for groups of organisms when features are selected on the group basis should be investigated.

Finally, all feature selection methods in this study, save PCF, are based on two classes and in the future additional feature selection methods that are independent of the negative class should be investigated. Since there is a large interest in the field of feature selection spawned by big data problems, many new methods have been devised and we plan to use some of them [41, 42] in future research to select a stable and performant set of features.

## Supplementary Material

Different feature selection methods were employed and the outcome was submitted to machine learning.
Supplementary File 1 contains the computational workflows detailing how feature selection was performed for the different methods. Figures 1 and 2 show training and testing schemes and figures 3 to 10 show the calculation workflow for the feature selection methods in this study.
Supplementary Table 1 contains the selected features and their information gain on a per feature selection algorithm basis (separated into work sheets) and on a per species basis (combined in one work sheet).
Supplementary Table 2 contains the classifier performance (RawData Sheet), Accuracy Plot for the individual selection methods (AccuracyFigure Sheet) and for the combined feature selection (Accuracy Sheet). Additional information like deviation among methods (Deviation Sheet), performance ranking (Sequence Sheet), and the construction of classifier performance (Comparison Sheet) are also provided.

## Figures and Tables

**Table 1:** Comparison of classification accuracies among this and other published methods (PlantMiRNAPred [32], Triplet-SVM [38], microPred [39], and MotifmiRNAPred [33]). The best performance per organism is highlighted in bold. ACC: accuracy.

Organism	PlantMiRNAPred	Triplet-SVM	microPred	MotifmiRNAPred	This study
	ACC	ACC	ACC	ACC	ACC
gma	**98.50**	74.10	86.70	89.80	97.38
zma	**98.30**	66.90	93.80	94.80	93.59
sbi	**98.40**	69.50	94.60	93.50	94.25
ath	92.20	76.00	89.40	93.30	**97.39**
ppt	92.40	71.40	89.50	90.20	**97.20**
ptc	91.80	75.20	84.90	92.20	**94.00**
osa	94.20	75.50	90.40	90.30	**95.32**
Avg	95.11	72.66	89.90	92.01	**95.59**

## References

[1] Lee R. C., Feinbaum R. L., Ambros V. (1993). The *C. elegans* heterochronic gene lin-4 encodes small RNAs with antisense complementarity to lin-14. *Cell*.

[2] Kim V. N., Han J., Siomi M. C. (2009). Biogenesis of small RNAs in animals. *Nature Reviews Molecular Cell Biology*.

[3] Tüfekci K. U., Öner M. G., Meuwissen R. L. J., Genç Ş. (2014). The role of microRNAs in human diseases. *Methods in Molecular Biology*.

[4] Chapman E. J., Carrington J. C. (2007). Specialization and evolution of endogenous small RNA pathways. *Nature Reviews Genetics*.

[5] Alural B., Duran G. A., Tufekci K. U. (2014). EPO mediates neurotrophic, neuroprotective, anti-oxidant, and anti-apoptotic effects via downregulation of miR-451 and miR-885-5p in SH-SY5Y neuron-like cells. *Frontiers in Immunology*.

[6] Alural B., Ozerdem A., Allmer J., Genc K., Genc S. (2015). Lithium protects against paraquat neurotoxicity by NRF2 activation and miR-34a inhibition in SH-SY5Y cells. *Frontiers in Cellular Neuroscience*.

[7] Zhang Z., Yu J., Li D. (2009). PMRD: plant microRNA database. *Nucleic Acids Research*.

[8] Griffiths-Jones S., Saini H. K., van Dongen S., Enright A. J. (2008). miRBase: tools for microRNA genomics. *Nucleic Acids Research*.

[9] Hsu S.-D., Lin F.-M., Wu W.-Y. (2011). MiRTarBase: a database curates experimentally validated microRNA-target interactions. *Nucleic Acids Research*.

[10] Yousef M., Jung S., Showe L. C., Showe M. K. (2008). Learning from positive examples when the negative class is undetermined—microRNA gene identification. *Algorithms for Molecular Biology*.

[11] Ding J., Zhou S., Guan J. (2010). MiRenSVM: towards better prediction of microRNA precursors using an ensemble SVM classifier with multi-loop features. *BMC Bioinformatics*.

[12] Wu Y., Wei B., Liu H., Li T., Rayner S. (2011). MiRPara: a SVM-based software tool for prediction of most probable microRNA coding regions in genome scale sequences. *BMC Bioinformatics*.

[13] Ritchie W., Gao D., Rasko J. E. J. (2012). Defining and providing robust controls for microRNA prediction. *Bioinformatics*.

[14] Saçar M. D., Allmer J. (2013). Current limitations for computational analysis of miRNAs in cancer. *Pakistan Journal of Clinical and Biomedical Research*.

[15] Saçar M. D., Hamzeiy H., Allmer J. (2013). Can MiRBase provide positive data for machine learning for the detection of MiRNA hairpins?. *Journal of Integrative Bioinformatics*.

[16] Yousef M., Najami N., Khalifav W. (2010). A comparison study between one-class and two-class machine learning for MicroRNA target detection. *Journal of Biomedical Science and Engineering*.

[17] Saçar M. D., Allmer J. (2014). Machine learning methods for microRNA gene prediction. *Methods in Molecular Biology*.

[18] Saçar M. D., Allmer J. Comparison of four Ab initio MicroRNA prediction tools.

[19] Ng K. L. S., Mishra S. K. (2007). De novo SVM classification of precursor microRNAs from genomic pseudo hairpins using global and intrinsic folding measures. *Bioinformatics*.

[20] Wei L., Liao M., Gao Y., Ji R., He Z., Zou Q. (2014). Improved and promising identificationof human microRNAs by incorporatinga high-quality negative set. *IEEE/ACM Transactions on Computational Biology and Bioinformatics*.

[21] Manevitz L. M., Yousef M. (2002). One-class SVMs for document classification. *Journal of Machine Learning Research*.

[22] Manevitz L., Yousef M. (2007). One-class document classification via Neural Networks. *Neurocomputing*.

[23] Yousef M., Allmer J., Khalifa W. (2016). Accurate plant MicroRNA prediction can be achieved using sequence motif features. *Journal of Intelligent Learning Systems and Applications*.

[24] Saçar M. D., Allmer J. Data mining for microrna gene prediction: on the impact of class imbalance and feature number for microrna gene prediction.

[25] Amaldi E., Kann V. (1998). On the approximability of minimizing nonzero variables or unsatisfied relations in linear systems. *Theoretical Computer Science*.

[26] Paul S., Magdon-Ismail M., Drineas P. (2015). Feature selection for linear SVM with provable guarantees. *Journal of Machine Learning Research*.

[27] Guyon I., Weston J., Barnhill S., Vapnik V. (2002). Gene selection for cancer classification using support vector machines. *Machine Learning*.

[28] Ahsen M. E., Singh N. K., Boren T., Vidyasagar M., White M. A. A new feature selection algorithm for two-class classification problems and application to endometrial cancer.

[29] Lorena L. H. N., Carvalho A. C. P. L. F., Lorena A. C. (2015). Filter feature selection for one-class classification. *Journal of Intelligent & Robotic Systems*.

[30] Xuan P., Guo M., Huang Y., Li W., Huang Y. (2011). Maturepred: efficient identification of microRNAs within novel plant Pre-miRNAs. *PLoS ONE*.

[31] Hall M., Frank E., Holmes G., Pfahringer B., Reutemann P., Witten I. H. (2009). The WEKA data mining software. *ACM SIGKDD Explorations Newsletter*.

[32] Xuan P., Guo M., Liu X., Huang Y., Li W., Huang Y. (2011). PlantMiRNAPred: efficient classification of real and pseudo plant pre-miRNAs. *Bioinformatics*.

[33] Yousef M., Allmer J., Khalifa W. (2015). Sequence motif-based one-class classifiers can achieve comparable accuracy to two-class learners for plant microRNA detection. *Journal of Biomedical Science and Engineering*.

[34] Saçar M. D., Bağcı C., Allmer J. (2014). Computational prediction of MicroRNAs from toxoplasma gondii potentially regulating the hosts' gene expression. *Genomics, Proteomics and Bioinformatics*.

[35] Tax D. M. J. (2015). *DDTools, the Data Description Toolbox for Matlab*.

[36] Berthold M. R., Cebron N., Dill F., Preisach C., Burkhardt H., Schmidt-Thieme L., Decker R. (2008). KNIME: the Konstanz Information Miner. *Data Analysis, Machine Learning and Applications*.

[37] Letunic I., Bork P. (2011). Interactive Tree of Life v2: online annotation and display of phylogenetic trees made easy. *Nucleic Acids Research*.

[38] Jiang P., Wu H., Wang W., Ma W., Sun X., Lu Z. (2007). MiPred: classification of real and pseudo microRNA precursors using random forest prediction model with combined features. *Nucleic Acids Research*.

[39] Batuwita R., Palade V. (2009). microPred: effective classification of pre-miRNAs for human miRNA gene prediction. *Bioinformatics*.

[40] Allmer J. (2014). Computational and bioinformatics methods for microRNA gene prediction. *Methods in Molecular Biology*.

[41] Zou Q., Zeng J., Cao L., Ji R. (2016). A novel features ranking metric with application to scalable visual and bioinformatics data classification. *Neurocomputing*.

[42] Zeng X., Liao Y., Liu Y., Zou Q. (2016). Prediction and validation of disease genes using HeteSim Scores. *IEEE/ACM Transactions on Computational Biology and Bioinformatics*.

