# Supplementary material for: Feature Selection Has a Large Impact on One-Class Classification Accuracy for MicroRNAs in Plants
Source: Adv Bioinformatics. 2016 Apr 12;2016:5670851. doi: 10.1155/2016/5670851 (PMC4844869; doi:10.1155/2016/5670851)
Supplement: Supplementary file 1 — Different feature selection methods were employed and the outcome was submitted to machine learning. Supplementary File 1 contains the computational workflows detailing how feature selection was performed for the different methods. Figures 1 and 2 show training and testing schemes and figures 3 to 10 show the calculation workflow for the feature selection methods in this study. Supplementary Table 1 contains the selected features and their information gain on a per feature selection algorithm basis (separated into work sheets) and on a per species basis (combined in one work sheet). Supplementary Table 2 contains the classifier performance (RawData Sheet), Accuracy Plot for the individual selection methods (AccuracyFigure Sheet) and for the combined feature selection (Accuracy Sheet). Additional information like deviation among methods (Deviation Sheet), performance ranking (Sequence Sheet), and the construction of classifier performance (Comparison Sheet) are also provided. [file 5670851.f1.zip › SupplementaryFile1.pptx]

## Slide 1
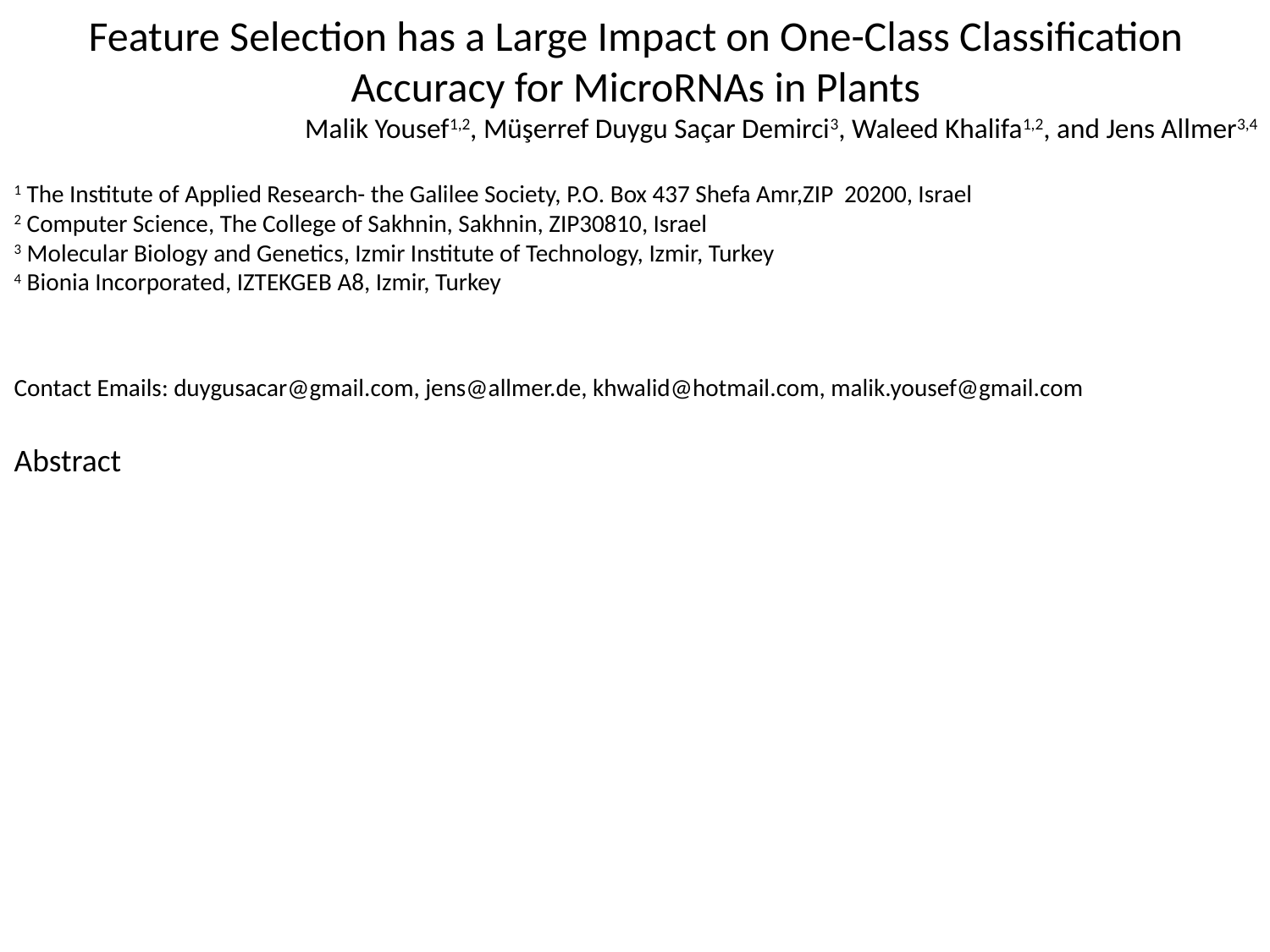

Feature Selection has a Large Impact on One-Class Classification Accuracy for MicroRNAs in Plants
Malik Yousef1,2, Müşerref Duygu Saçar Demirci3, Waleed Khalifa1,2, and Jens Allmer3,4
1 The Institute of Applied Research- the Galilee Society, P.O. Box 437 Shefa Amr,ZIP 20200, Israel
2 Computer Science, The College of Sakhnin, Sakhnin, ZIP30810, Israel
3 Molecular Biology and Genetics, Izmir Institute of Technology, Izmir, Turkey
4 Bionia Incorporated, IZTEKGEB A8, Izmir, Turkey
Contact Emails: duygusacar@gmail.com, jens@allmer.de, khwalid@hotmail.com, malik.yousef@gmail.com
Abstract

## Slide 2
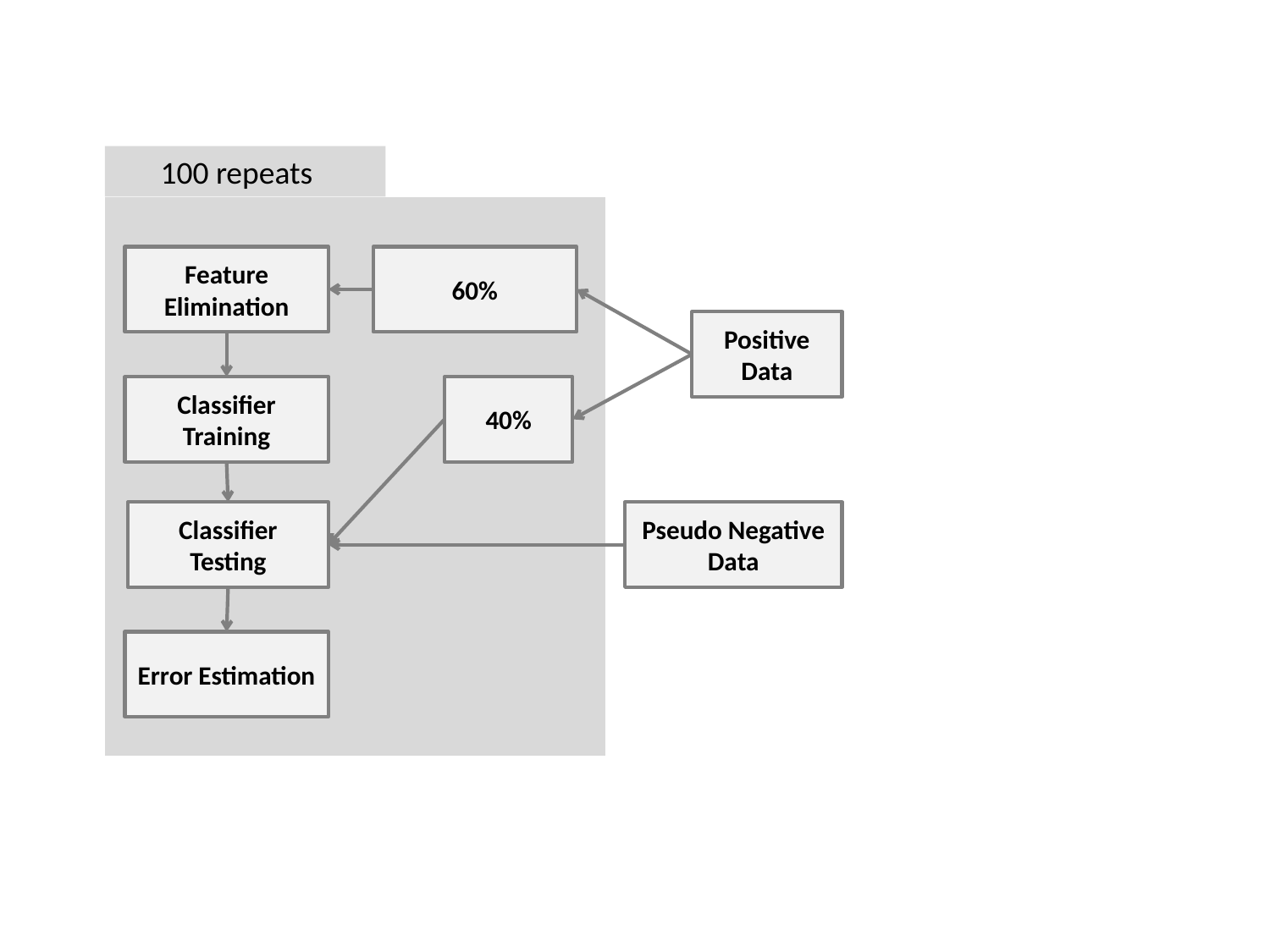

100 repeats
Feature Elimination
60%
Positive Data
Classifier Training
40%
Classifier Testing
Pseudo Negative Data
Error Estimation

## Slide 3
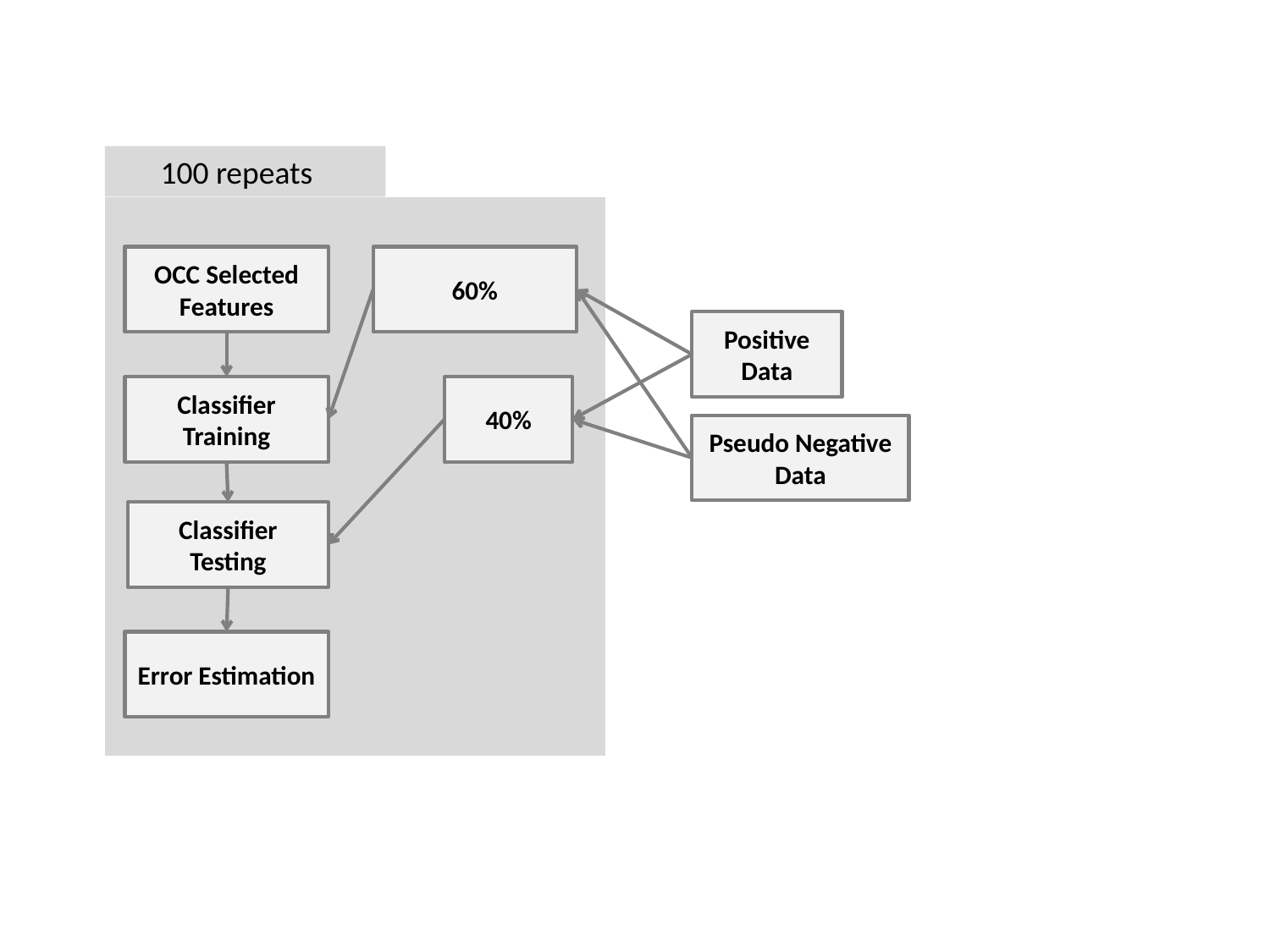

100 repeats
OCC Selected Features
60%
Positive Data
Classifier Training
40%
Pseudo Negative Data
Classifier Testing
Error Estimation

## Slide 4
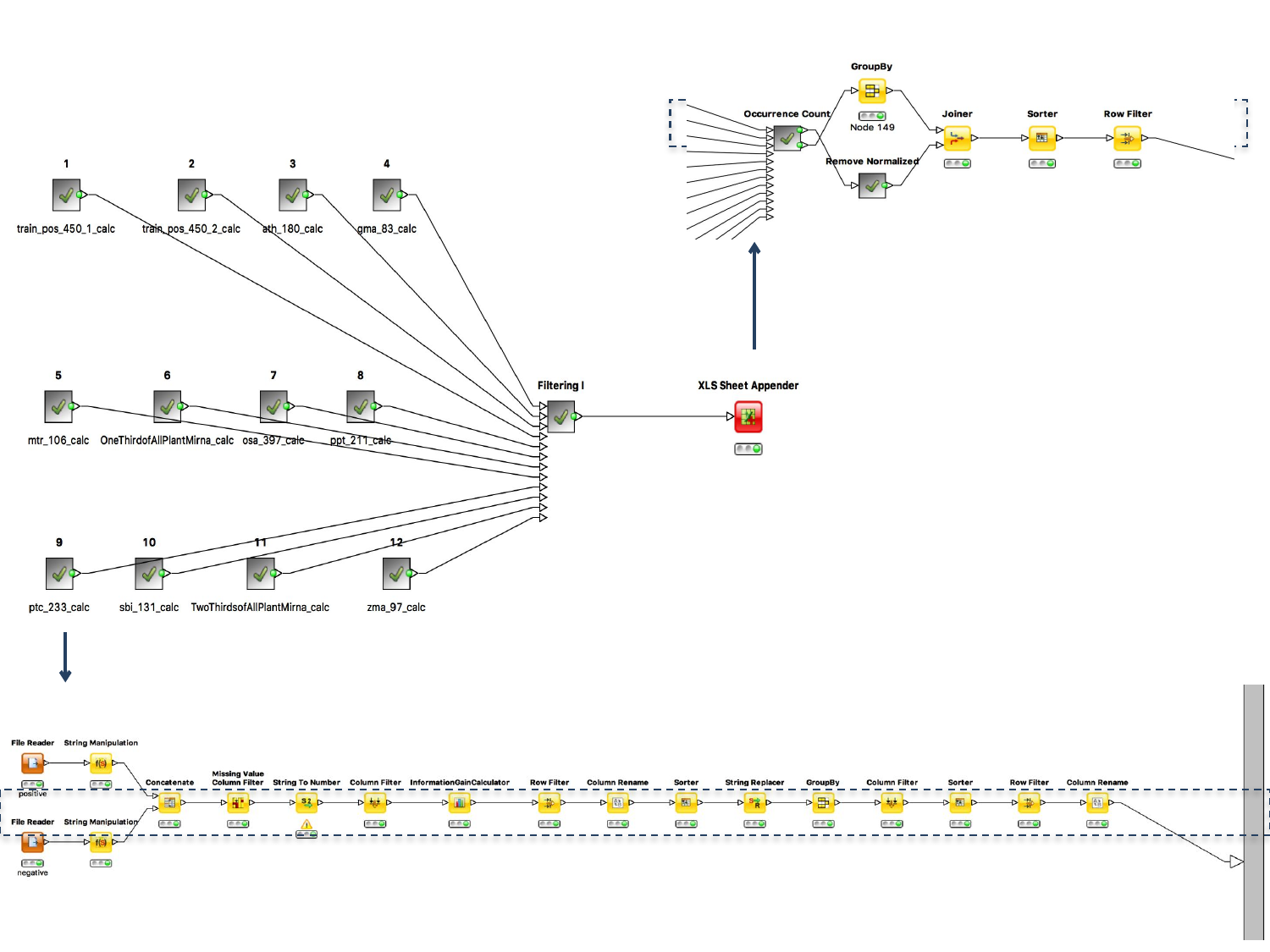

## Slide 5
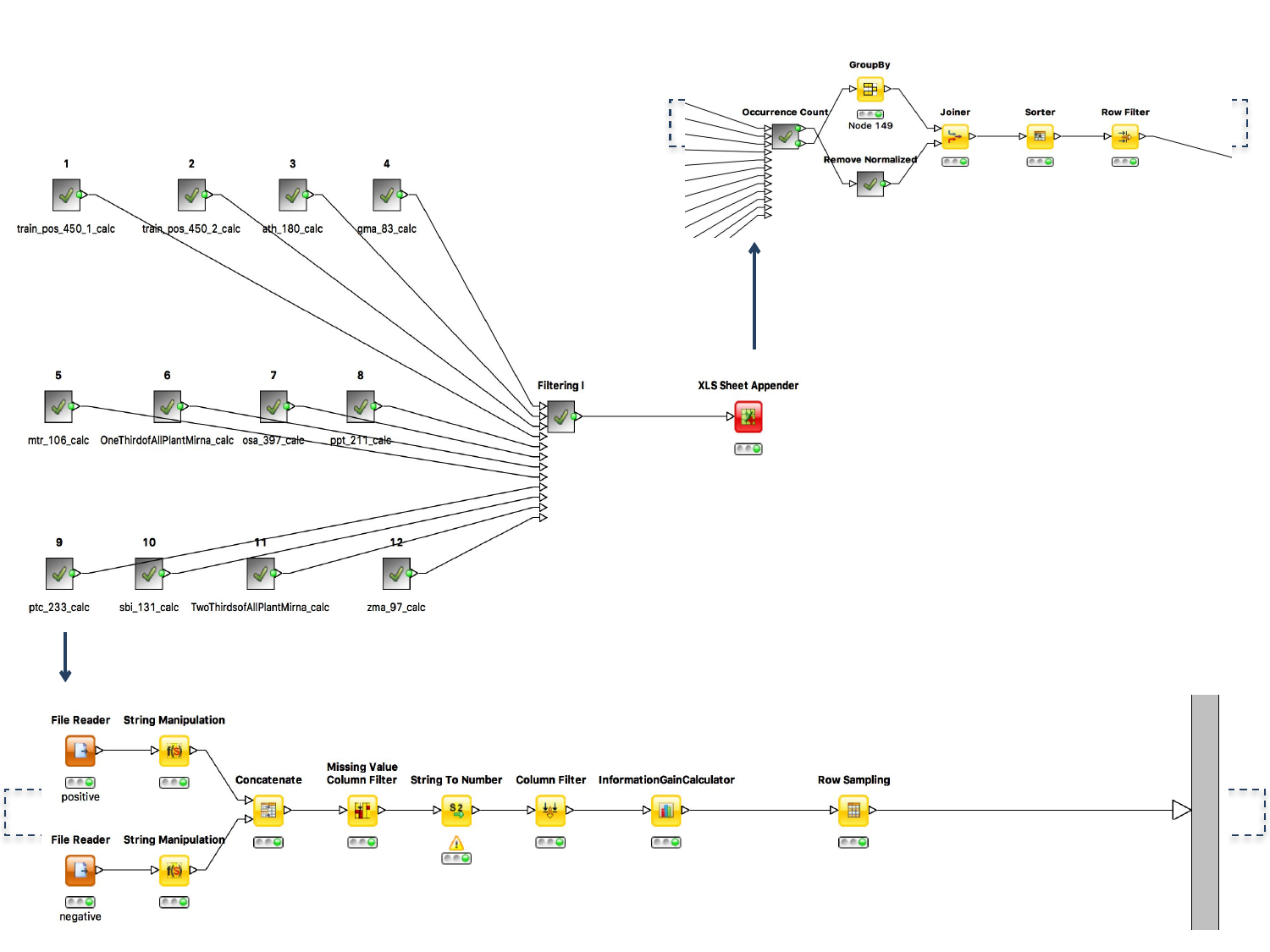

## Slide 6
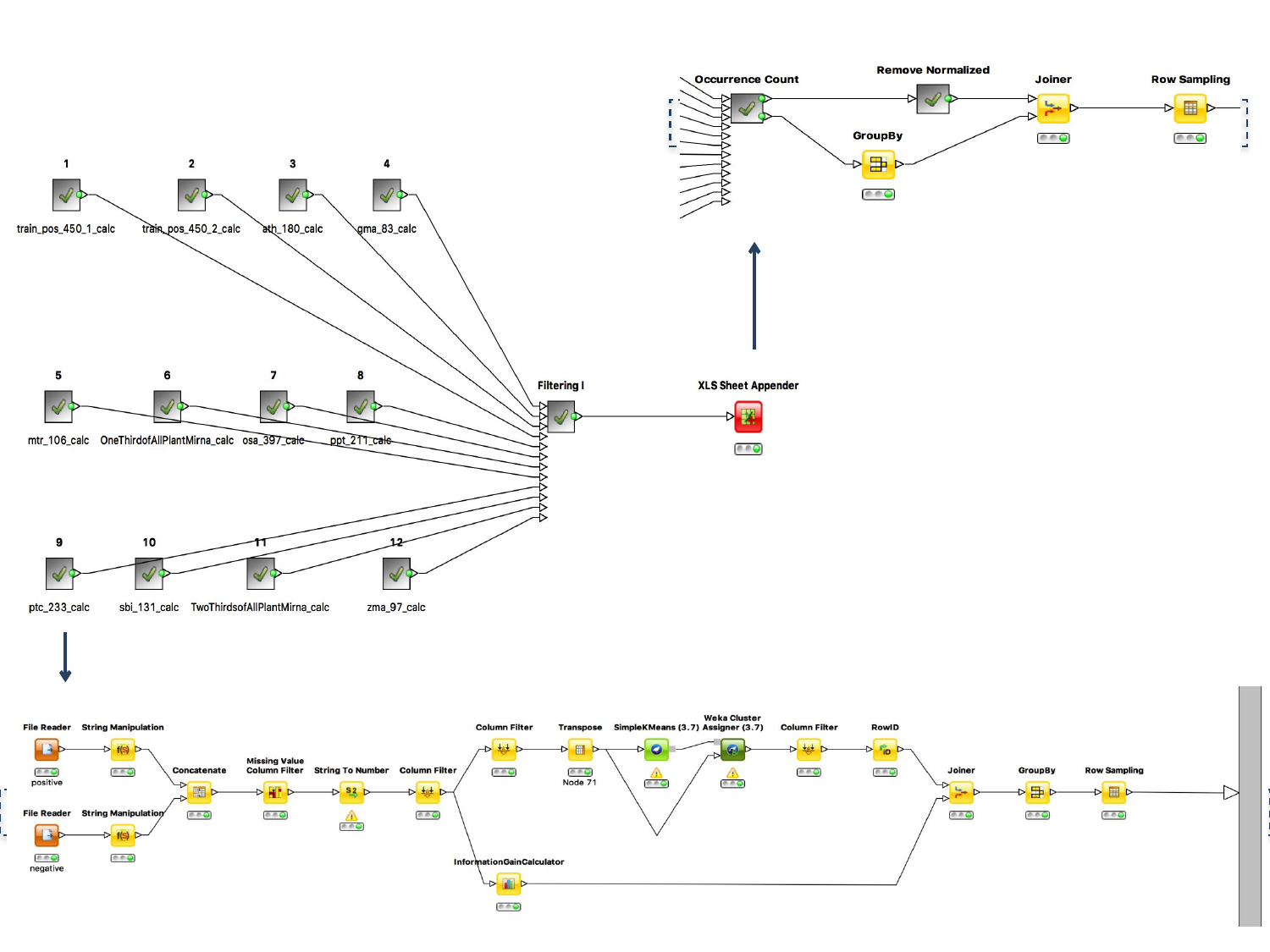

## Slide 7
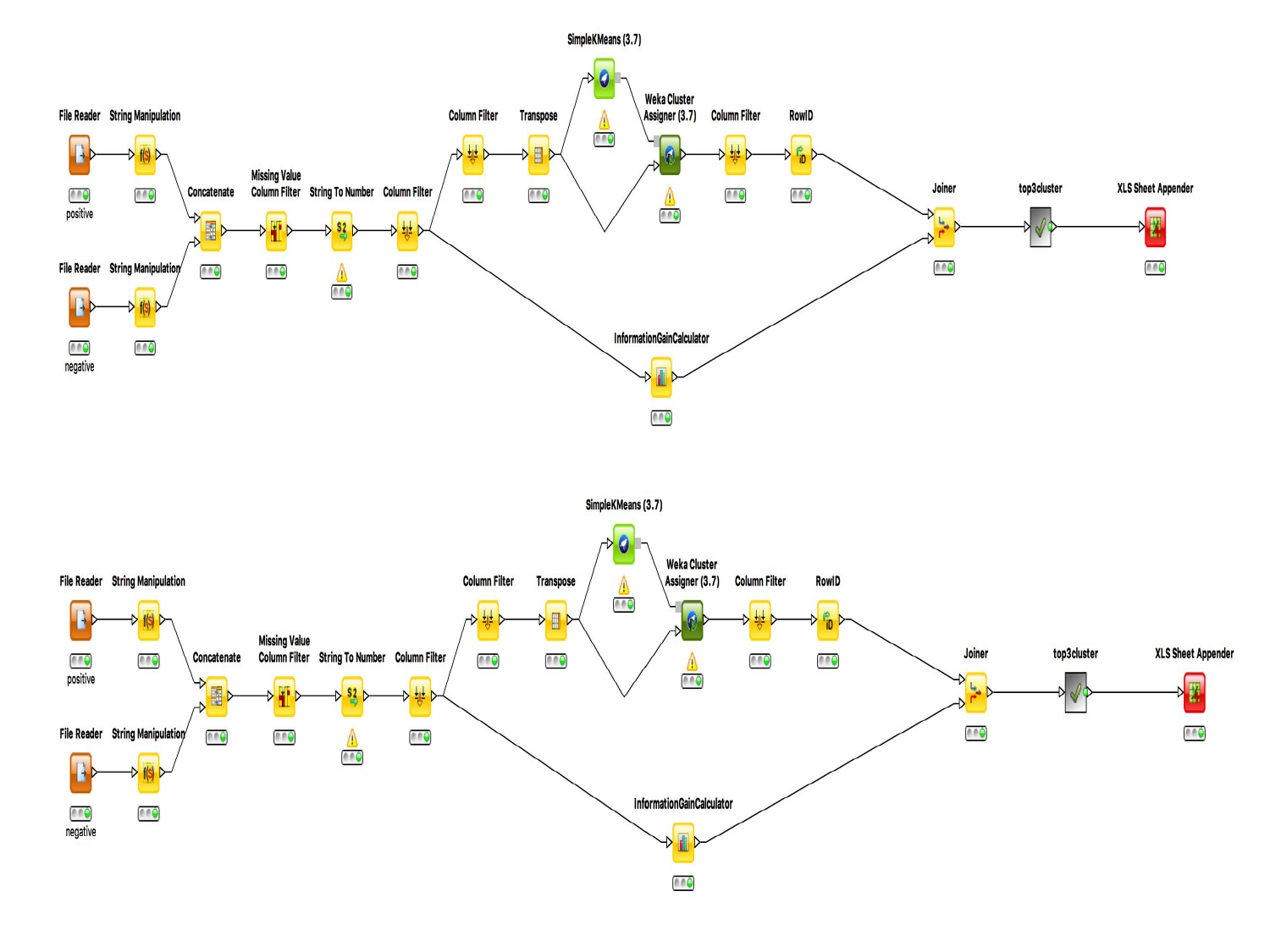

## Slide 8
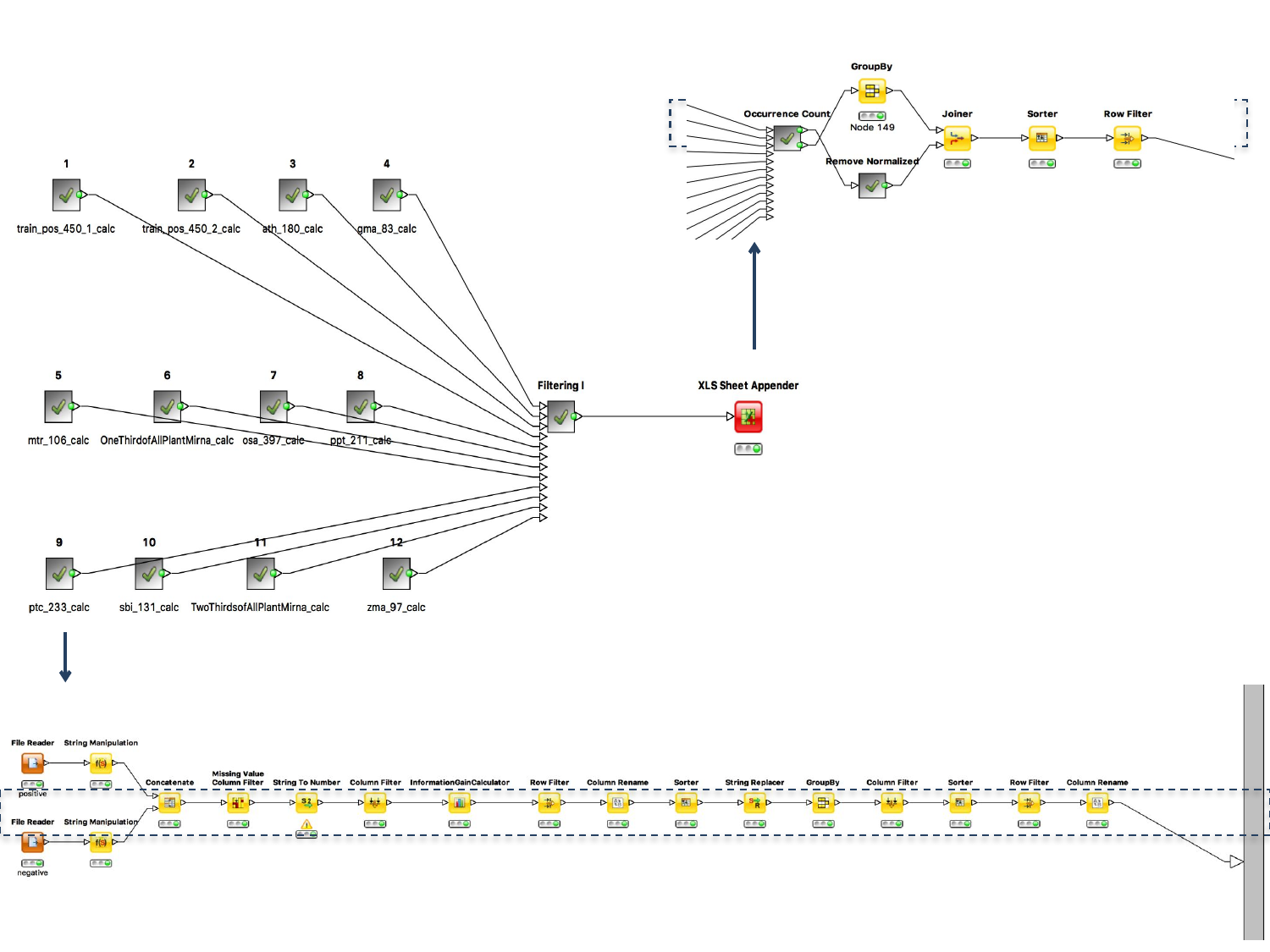

## Slide 9
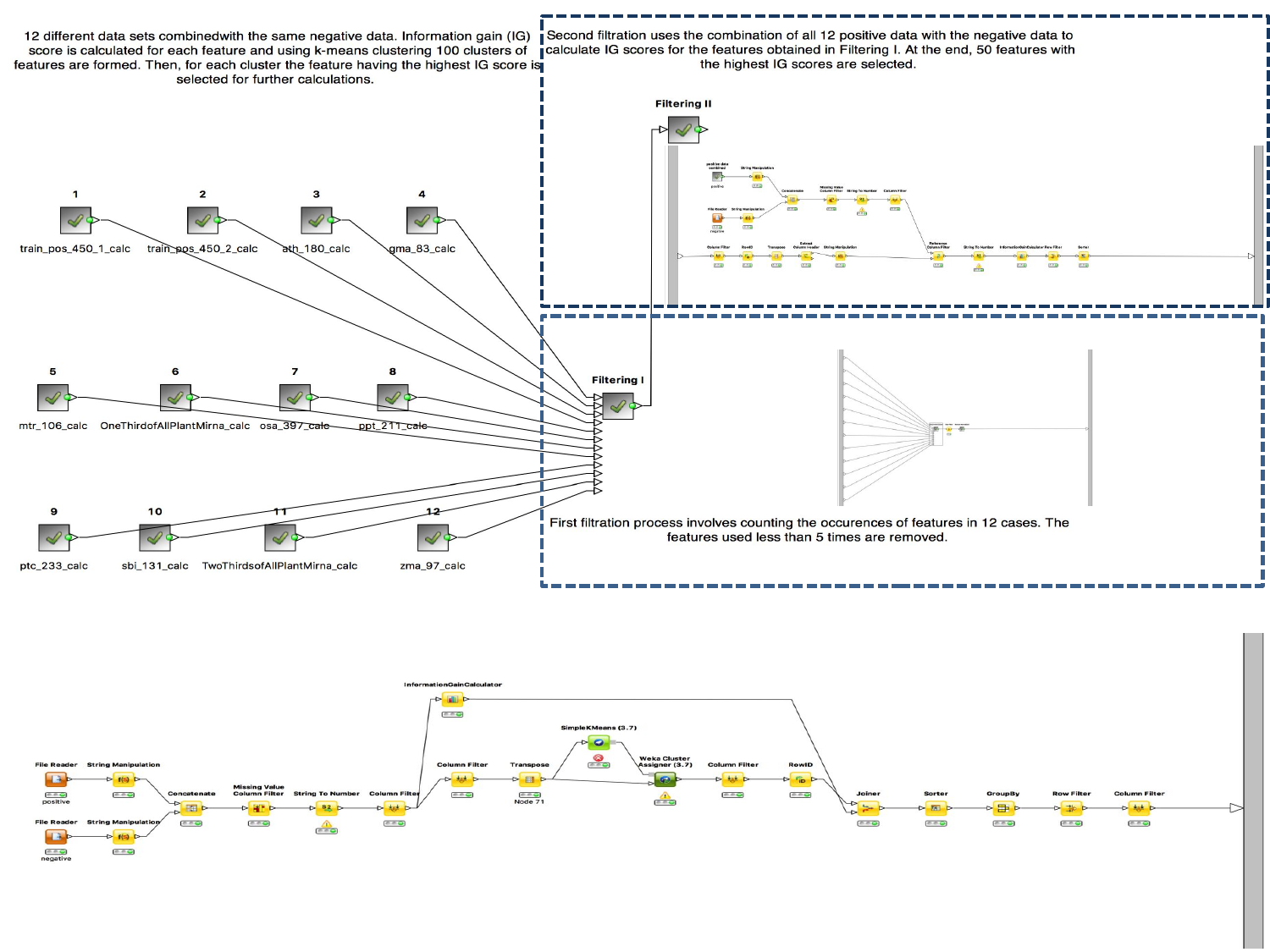

## Slide 10
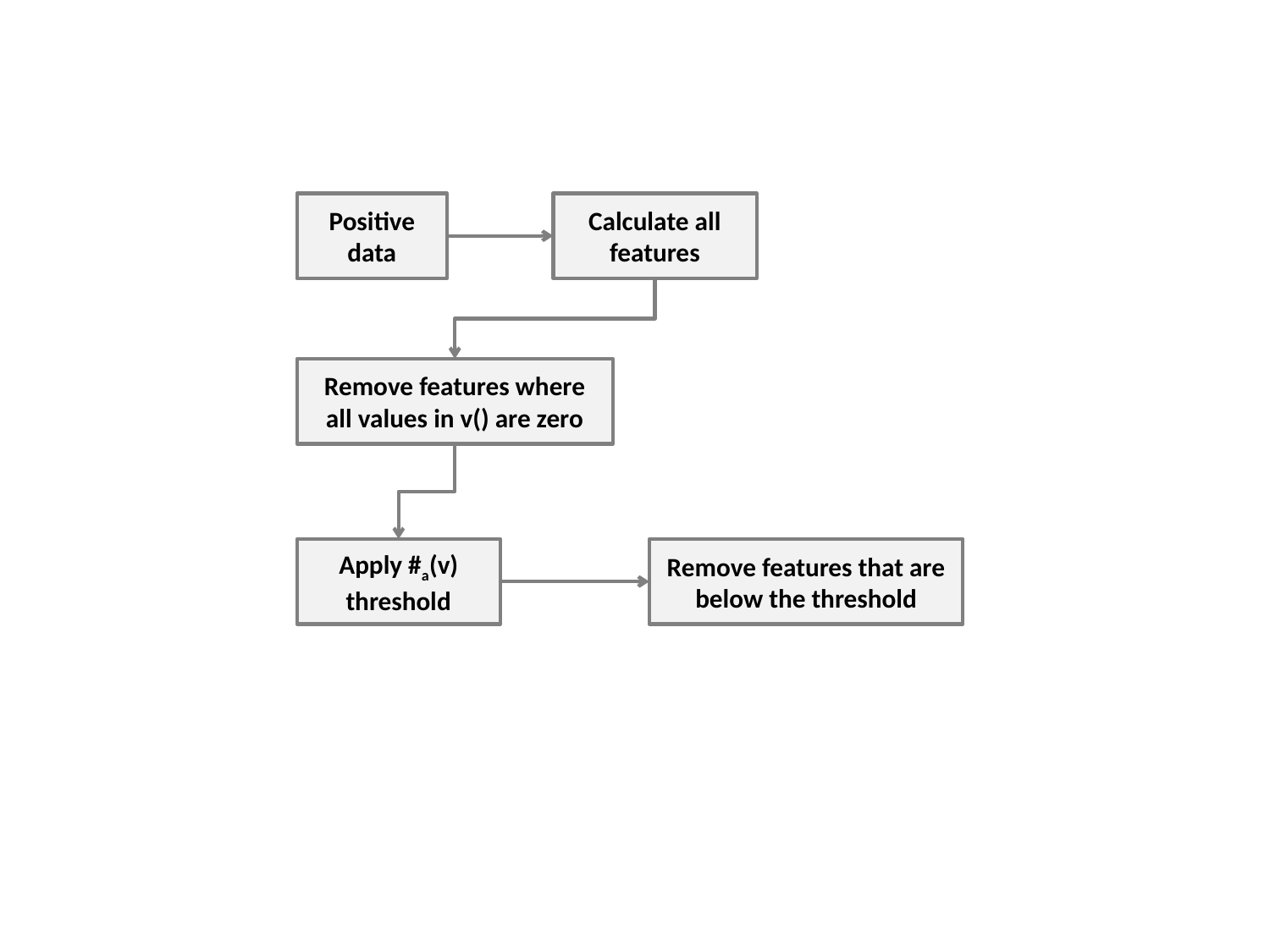

Positive data
Calculate all features
Remove features where all values in v() are zero
Apply #a(v) threshold
Remove features that are below the threshold

## Slide 11
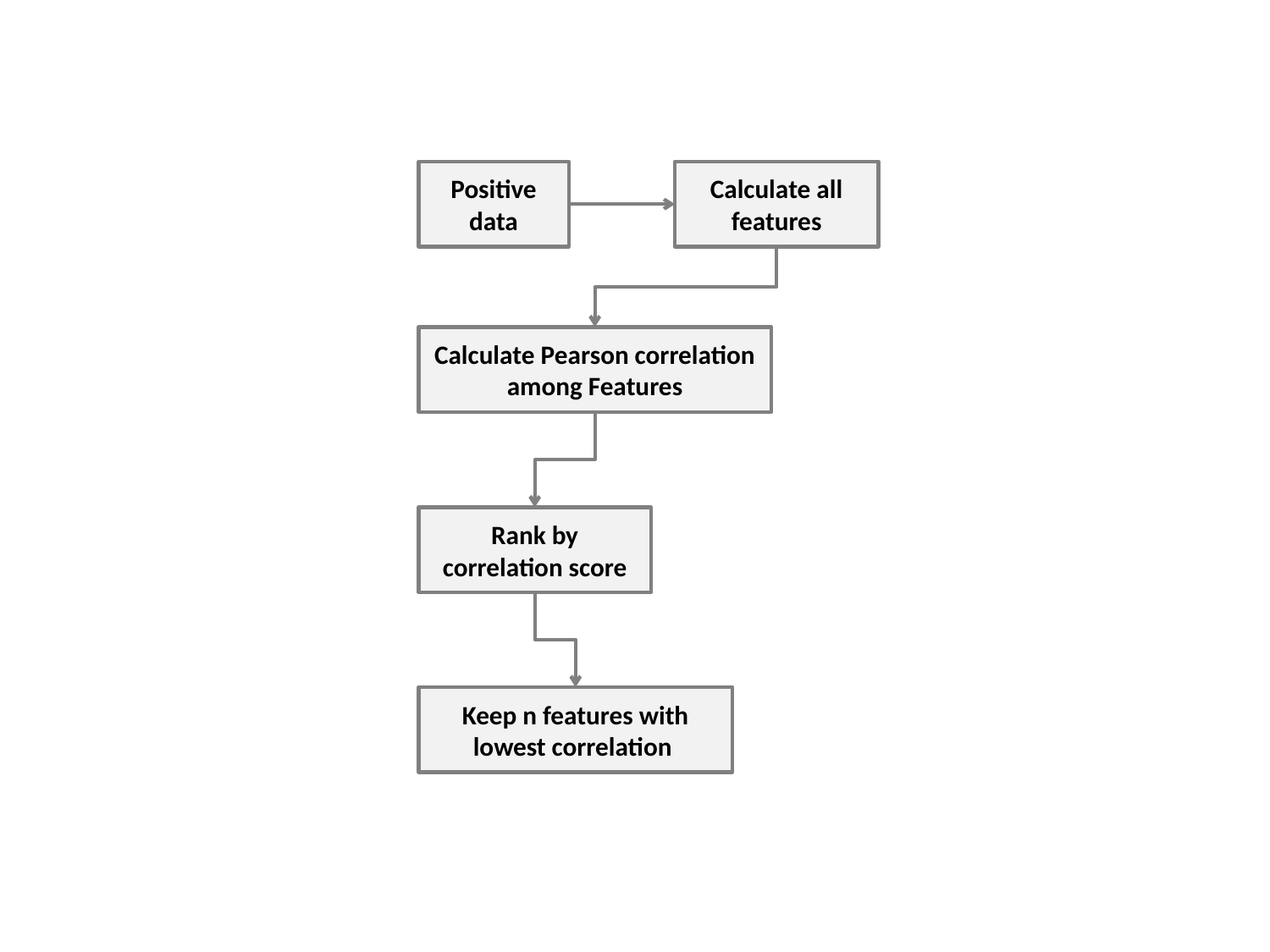

Positive data
Calculate all features
Calculate Pearson correlation among Features
Rank by correlation score
Keep n features with lowest correlation
