# Supplementary figures and images for: Feature Selection Has a Large Impact on One-Class Classification Accuracy for MicroRNAs in Plants
Source: Adv Bioinformatics. 2016 Apr 12;2016:5670851. doi: 10.1155/2016/5670851 (PMC4844869; doi:10.1155/2016/5670851)

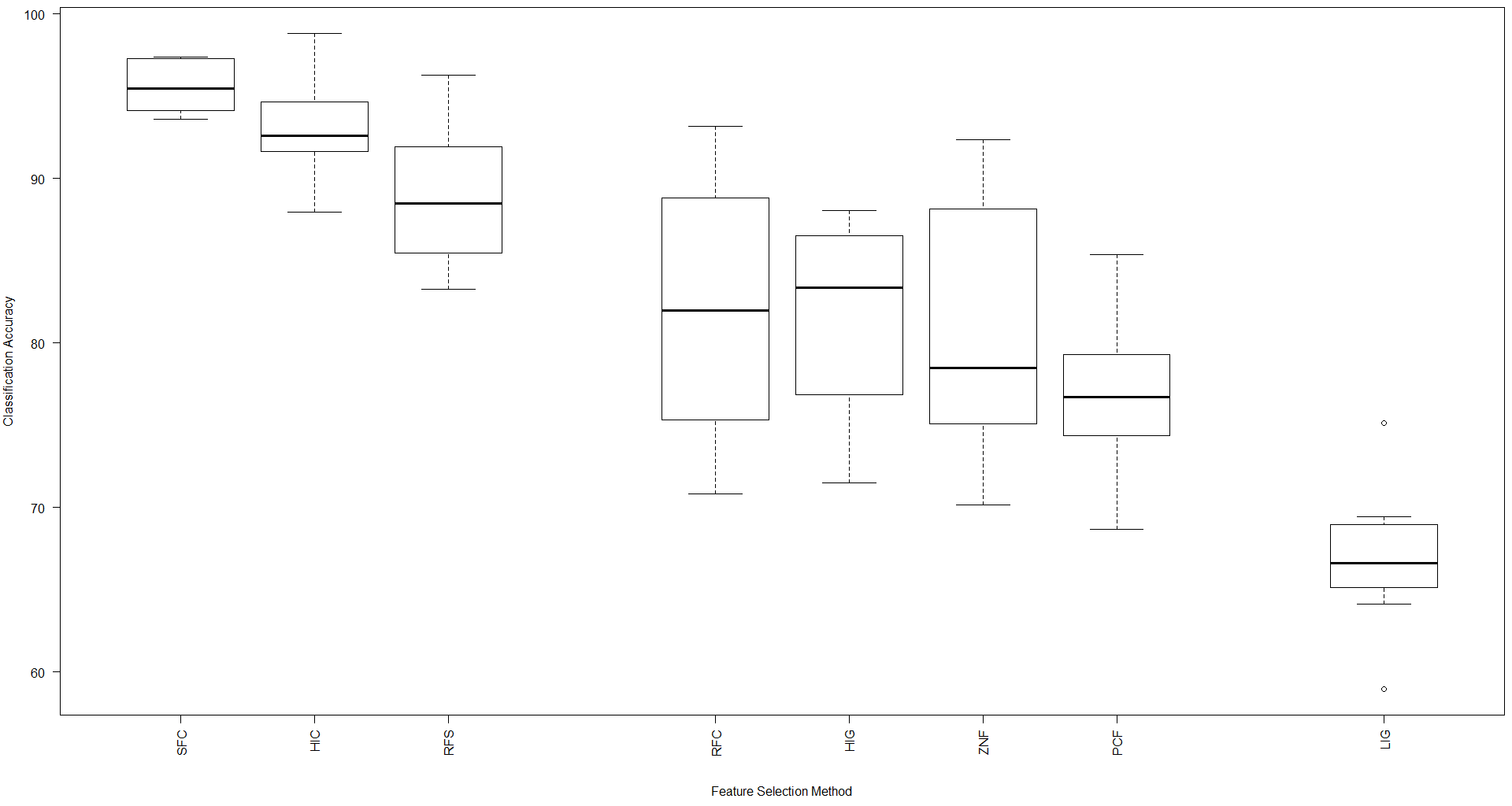

Supplement: Supplementary file 1 — Different feature selection methods were employed and the outcome was submitted to machine learning. Supplementary File 1 contains the computational workflows detailing how feature selection was performed for the different methods. Figures 1 and 2 show training and testing schemes and figures 3 to 10 show the calculation workflow for the feature selection methods in this study. Supplementary Table 1 contains the selected features and their information gain on a per feature selection algorithm basis (separated into work sheets) and on a per species basis (combined in one work sheet). Supplementary Table 2 contains the classifier performance (RawData Sheet), Accuracy Plot for the individual selection methods (AccuracyFigure Sheet) and for the combined feature selection (Accuracy Sheet). Additional information like deviation among methods (Deviation Sheet), performance ranking (Sequence Sheet), and the construction of classifier performance (Comparison Sheet) are also provided. [file 5670851.f1.zip › AccuracyDistribution.png]

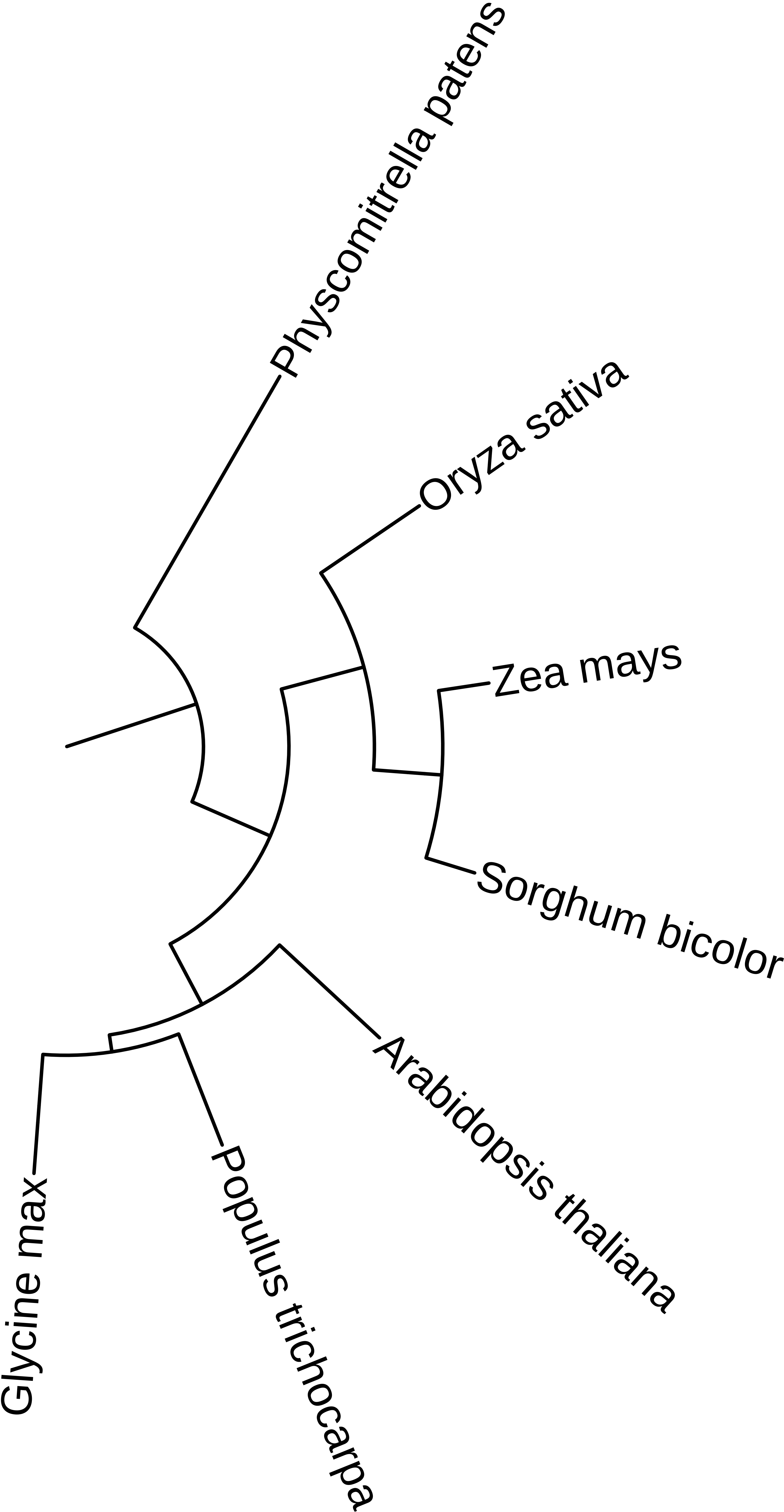

Supplement: Supplementary file 1 — Different feature selection methods were employed and the outcome was submitted to machine learning. Supplementary File 1 contains the computational workflows detailing how feature selection was performed for the different methods. Figures 1 and 2 show training and testing schemes and figures 3 to 10 show the calculation workflow for the feature selection methods in this study. Supplementary Table 1 contains the selected features and their information gain on a per feature selection algorithm basis (separated into work sheets) and on a per species basis (combined in one work sheet). Supplementary Table 2 contains the classifier performance (RawData Sheet), Accuracy Plot for the individual selection methods (AccuracyFigure Sheet) and for the combined feature selection (Accuracy Sheet). Additional information like deviation among methods (Deviation Sheet), performance ranking (Sequence Sheet), and the construction of classifier performance (Comparison Sheet) are also provided. [file 5670851.f1.zip › phylogeny2.png]
